# Retinal Epithelial Neutralization Assay Optimizes AAV Serotype Selection for Ocular Gene Therapy

**DOI:** 10.3390/v17070988

**Published:** 2025-07-15

**Authors:** Yao Li, Yujia Chen, Nan Huo, Zuyuan Jia, He Huang, Zhenghao Zhao, Shipo Wu, Lihua Hou

**Affiliations:** Laboratory of Advanced Biotechnology, Beijing Institute of Biotechnology, Beijing 100071, China; yaoli71@163.com (Y.L.); frchem@163.com (Y.C.); huonanac@yeah.net (N.H.); chiazy20@zju.edu.cn (Z.J.); to-ni_kr8s@163.com (H.H.)

**Keywords:** adeno-associated virus (AAV), neutralizing antibody (NAb), serotype-dependent hierarchy, retinal gene therapy

## Abstract

Adeno-associated virus (AAV) vectors face a critical translational challenge in ocular gene therapy due to pre-existing neutralizing antibodies (NAbs) whose seroprevalence limits patient eligibility. Standard NAb detection using non-ocular cell models (Human Embryonic Kidney 293T) may inadequately predict retinal transduction inhibition due to cell type-related variations in receptor usage and immunogenicity. This study established parallel NAb detection platforms utilizing human retinal pigment epithelial (ARPE-19) cells and standard 293T cells to systematically evaluate clinical serum samples against ophthalmologically relevant AAV serotypes (2, 5, 8, 9) via luciferase reporter-based transduction inhibition assays. Comparative analysis demonstrated ARPE-19 exhibited 42–48% higher NAb titers against AAV5/9 compared to 293T cells, with distinct serotype-biased neutralization hierarchies observed between cellular models. Furthermore, female-derived sera exhibited significantly elevated NAbs against particular serotypes in the ARPE-19 system. Critically, inter-serotype cross-neutralization correlation patterns differed substantially between cellular platforms. These findings demonstrate that physiologically relevant retinal cellular models provide essential immunological profiling data, revealing NAb characteristics obscured in standard assays. Consequently, employing retinal cell-based platforms is crucial for optimizing AAV serotype selection, patient stratification, and predicting clinical outcomes in ocular gene therapy.

## 1. Introduction

AAV has emerged as a preeminent gene therapy vector for ophthalmic diseases, primarily attributable to its distinctive biological characteristics encompassing long-term sustained transgene expression profiles, minimal immunogenicity, and precise tissue-targeting capabilities [1]. Current ophthalmic research utilizes different AAV serotypes, each with unique tissue targeting and efficiency, allowing tailored treatments for various eye disorders [2,3,4,5].

Despite advancements in AAV-based therapies, their clinical implementation faces significant hurdles, notably the inhibitory effects of pre-existing NAbs in patients, which have emerged as a critical limiting factor [6,7,8,9]. Epidemiological studies indicated seroprevalence rates of AAV-specific NAbs ranging from 30 to 60% across various serotypes [10,11], significantly reducing the pool of patients eligible for treatment. This immunological barrier primarily stems from prior wild-type AAV exposure, which diminishes therapeutic accessibility for genetically targeted interventions. In ocular gene therapy, while localized subretinal injection administration partially circumvents systemic immune responses [12], emerging evidence indicates that intraocular NAb presence may still compromise therapeutic outcomes through vector neutralization in the targeted microenvironment [13]. These findings underscore the necessity for comprehensive immunological profiling, including the precise quantification of ocular NAb titers and functional assessment of their cell type-specific inhibitory effects, to optimize retinal gene therapy protocols and enhance clinical efficacy.

The current methodology for detecting neutralizing antibodies primarily depends on in vitro neutralization assays. The conventional protocol involves incubating patient serum with AAV vectors that encode reporter genes, such as luciferase or green fluorescent protein. This is followed by quantifying neutralizing antibody titers by measuring the levels of transduction inhibition in target cells, specifically 293T cells [14]. However, this approach presents critical limitations. AAV exhibits cell type-dependent receptor utilization during transduction [15,16,17]; for example, heparan sulfate proteoglycan (HSPG) serves as the primary receptor for AAV2 in most cellular contexts [18], yet enzymatic HSPG digestion paradoxically enhances AAV2 retinal transduction efficiency [19]. Such receptor-specific variations could compromise antibody–virus binding efficiency, thereby rendering 293T-based assessments insufficient for predicting actual AAV transduction inhibition in retinal tissues. Consequently, establishing retinal cell-based neutralization platforms holds significant potential for enhancing preclinical predictive value in retinal degeneration studies and optimizing patient stratification protocols.

This study established neutralizing antibody detection platforms using ARPE-19 and 293T cell lines. A comparative analysis of neutralizing antibody levels against ophthalmologically relevant AAV serotypes (2/5/8/9) in 300 clinical serum samples provided critical insights for optimizing AAV serotype selection in ocular gene therapy.

## 2. Materials and Methods

### 2.1. Recombinant AAV Production

We constructed a DNA cassette containing the luciferase gene regulated by the CMV promoter and polyA sequence, flanked by the internal terminal repeats of pAAV-CMV (Takara, Dalian, China, 6230). All AAV vectors were packaged in 293T cells, which were seeded in a 15 cm culture dish. Transfection was performed the following day, once cell confluence reached 80%, using polyethyleneimine (PEI) (Serochem, Atlanta, GA, USA, PRIME-P100) at a weight ratio of 3:1 with DNA. Additionally, AAV vectors were generated via triple plasmid transfection of 293T cells, wherein the pHelper plasmid (Takara, Dalian, China, 6230), pRep-rCap plasmid (pRep-Cap2: Takara, Dalian, China, 6230; pRep-Cap5: HonorGene, Beijing, China, HG-VXH1155; pRep-Cap8: HonorGene, Beijing, China, HG-VXH1156; pRep-Cap9: HonorGene, Beijing, China, HG-VXH1157), and ITR-containing plasmid were transfected at a molar ratio of 2:1:1 using PEI.

### 2.2. Human Serum Samples

This retrospective epidemiological investigation included participants enrolled in clinical studies at the Beijing Institute of Biotechnology, China, between 2019 and 2023. The serum samples stored in the institute’s biobank, originally collected for unrelated clinical trials, were analyzed. A randomized sampling strategy was implemented to achieve a target cohort of 300 individuals (113 females, 187 males), aged 18–70 years (Table 1).

### 2.3. Ethical Statement

All human serum samples utilized in this investigation were obtained in accordance with ethical guidelines established by the Ethics Committee of Academy of Military Medical Sciences.

### 2.4. Neutralizing Antibody Assay

Optimal multiplicity of infection (MOI) for AAVs was determined by correlating gradient MOIs (10^0^–10^5^) with luciferase activity in 239T and ARPE-19 cells. Cells were digested, resuspended in DMEM containing 10% FBS to 1 × 10^5^ cells/mL, and seeded into 96-well plates (100 μL/well). AAVs diluted in DMEM (10% FBS) were added (100 μL/well) to infect cells, followed by 24 h incubation (37 °C, 5% CO_2_) prior to luciferase quantification.

During the analytical process, serum samples were subjected to heat inactivation at 56 °C for 1 h. NAb activity targeting AAV2, AAV5, AAV8, and AAV9 serotypes was quantitatively evaluated through a standardized cell-based transduction inhibition assay. More specifically, recombinant AAV vectors carrying the firefly luciferase reporter gene, prepared at a MOI of 10^5^, were pre-incubated with diluted serum samples (dilution ratios of 1:2.5, 1:40, 1:160, 1:640, and 1:2560) for 1 h at 37 °C before being administered to cells. Subsequently, the 293T or ARPE-19 cells were seeded in 96-well plates at a density of 1 × 10^4^ cells/well and incubated under 5% CO_2_ at 37 °C. Luciferase activity quantification was performed in strict accordance with the manufacturer’s protocol (Promega, Madison, WI, USA, E1501) using a GloMax Navigator microplate luminometer (Promega, Madison, WI, USA, GM2010). NAb activity was quantified by luciferase signal attenuation 24 h post-infection, demonstrating the dose-dependent inhibition of functional vector transduction efficiency. Neutralizing titers (NT_90_) were calculated as the serum dilution achieving 90% viral neutralization through four-parameter logistic regression modeling of neutralization percentages against antibody dilution series. Valid assays required ≥80% reporter activity in virus-only controls versus untreated cells and sigmoidal dose–response curves (R^2^ ≥ 0.9) in test samples.

### 2.5. Statistical Analysis

All statistical analyses in this study were performed using GraphPad Prism software (version 8.0.2, GraphPad Inc., San Diego, CA, USA). The data are reported as the geometric mean ± geometric standard deviation. Comparisons between two groups were evaluated with the paired or unpaired *t* test, while comparisons among three or more groups were conducted using one-way analysis of variance (ANOVA). When the distributional assumptions for parametric tests were violated in paired data analyses, the Wilcoxon signed-rank test was implemented to evaluate the significance of median differences.

## 3. Results

### 3.1. Overall Neutralizing Antibody Profiles

**Figure 1:**
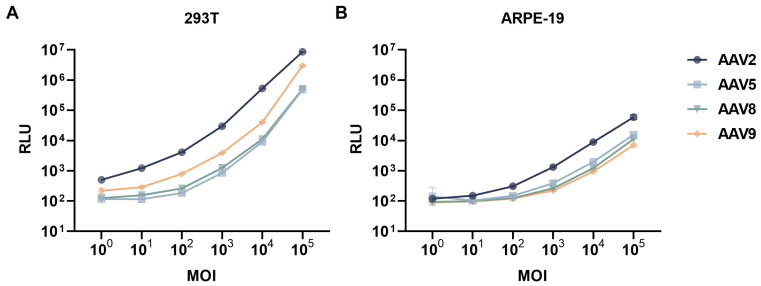
Dose-dependent transduction efficiency of AAV serotypes in 293T and ARPE-19 cells. Dose–response profiles of AAV2, AAV5, AAV8, and AAV9 infections in 293T cells (**A**) and ARPE-19 cells (**B**) at varying MOI. The dose-dependent curves quantify serotype-specific transduction efficiency disparities, reflecting their distinct cellular tropisms mediated by receptor binding specificity.

Systematic dose–response profiling demonstrated a statistically significant linear correlation between MOI and normalized luciferase activity in both 293T and ARPE-19 cellular models at MOI = 10^3^–10^5^ (R^2^ > 0.95) (Figure 1A,B). This optimized parameter of MOI = 10^5^ was subsequently implemented in all neutralization assays to ensure standardized transduction efficiency across experimental conditions. We employed the NT_90_ (normalized concentration threshold at 90% detection efficiency) method to quantify NAb titers against diverse AAV serotypes in two cellular systems.

**Figure 2:**
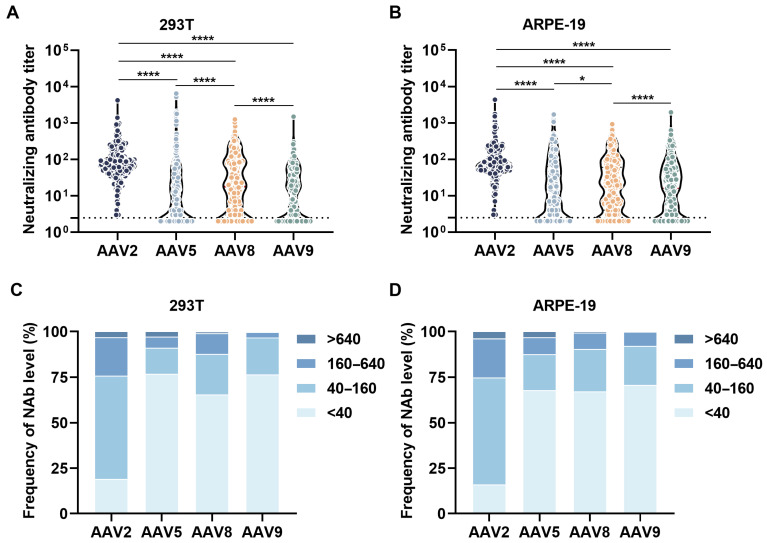
Neutralization profiles and serotype-specific seroprevalence of AAV in human sera. (**A**,**B**) Neutralizing activity (NT_90_) against AAV2/5/8/9 in human sera assessed by in vitro transduction inhibition in 293T (**A**) and ARPE-19 (**B**) cells. The NAb titers, quantified through RLU reduction measurements of pre-incubated AAV-luciferase complexes, revealed distinct serotype-specific antibody neutralization profiles across cellular models. (**C**,**D**) Histograms present comparative data for AAV2, AAV5, AAV8, and AAV9 corresponding to the experimental results shown in (**A**,**B**), systematically illustrating the distribution patterns of serum NAb against each AAV serotype. *p*-value calculated between two groups by paired *t*-test; data are presented as GMT with GSD (*n* = 3); * *p* < 0.05; **** *p* < 0.0001.

In 293T cellular assays, serum samples demonstrated a serotype-dependent neutralization hierarchy (Figure 2A,C). Anti-AAV2 NAbs exhibited the highest prevalence, displaying a geometric mean titer (GMT) with geometric standard deviation (GSD) of 77.8 ± 3.0 across all analyzed specimens (Table 2). Notably, the proportion of samples demonstrating AAV2 NAb titers below the critical threshold of 1:40 dilution showed a statistically significant reduction compared to other AAV serotypes. Conversely, anti-AAV5 NAbs manifested comparatively reduced neutralizing capacity within this experimental framework. Quantitative stratification revealed a descending gradient of neutralization efficiency: AAV9 (10.1 ± 4.6) < AAV5 (10.3 ± 6.0) < AAV8 (17.7 ± 5.5).

Notably, distinct neutralization profiles emerged in ARPE-19 cellular assays, with system-wide inhibition rates being markedly attenuated relative to 293T counterparts (Figure 2B,D). While AAV2 remained the most susceptible serotype (GMT of 79.1), the hierarchical ranking of serum-mediated repression demonstrated differences among other serotypes, AAV9 < AAV5 < AAV8, with corresponding GMT ± GSD values of 14.3 ± 5.0 (AAV9, 42% increase versus 293T), 15.3 ± 6.5 (AAV5, 48% increase versus 293T), and 16.8 ± 5.0 (AAV8). Crucially, comparative analysis revealed that NAb levels against AAV5 quantified through ARPE-19 detection systems significantly exceeded those measured in 293T cells.

### 3.2. Age-Stratified Neutralizing Antibody Profiles

**Figure 3:**
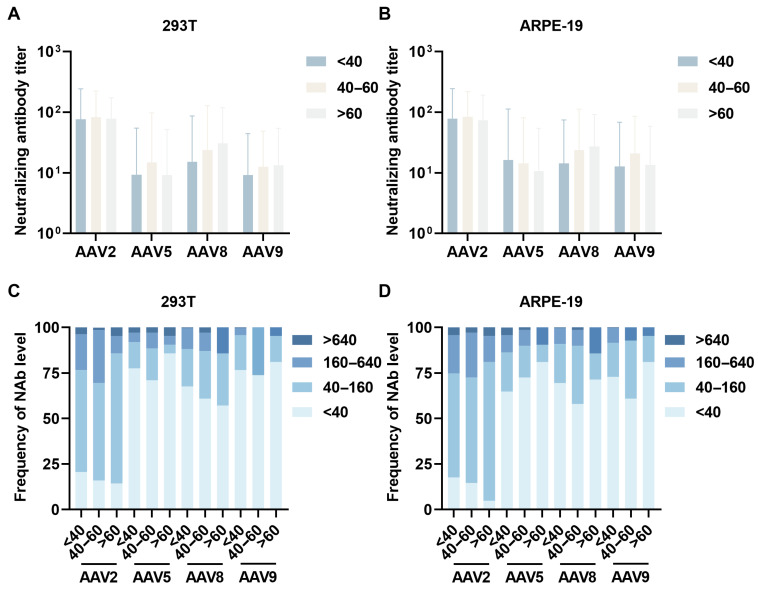
Age-stratified NAb profiles. Neutralizing antibody levels from Figure 2 were stratified by serum donor age into three cohorts: <40 years, 40–60 years, and >60 years. NAb titers against wild-type AAV2, AV5, AAV8, and AAV9 were assayed in 293T cells (**A**) and ARPE-19 cells (**B**), revealing age-dependent variations in serotype-specific neutralization capacity. (**C**,**D**) Histograms present comparative data for AAV2, AAV5, AAV8, and AAV9 corresponding to the experimental results shown in (**A**,**B**), systematically illustrating the distribution patterns of serum NAb across age groups against each AAV serotype. Statistical analysis calculated between two serotypes by unpaired *t*-test; data are presented as GMT with GSD.

Participants were stratified into three age cohorts (<40 years, 40–60 years, and >60 years) to systematically evaluate age-dependent variations in NAb titers against AAV serotypes. Quantitative sero-epidemiological analysis revealed distinct age-specific NAb profiles, with the 40–60 years cohort demonstrating significantly elevated GMT against AAV2 (83.0 ± 2.7) and AAV5 (14.7 ± 6.6) compared to younger (<40 years: AAV2 76.1 ± 3.2, AAV5 9.3 ± 5.8) and older (>60 years: AAV2 78.0 ± 2.2, AAV5 9.1 ± 5.6) cohorts, suggesting a bell-shaped seroprevalence curve with maximal exposure probability during middle adulthood. For AAV8 and AAV9, we observed progressive age-associated increases in NAb titers, with elderly cohorts exhibiting 2-fold higher neutralization capacity against AAV8 compared to the youngest group (GMT: 30.6 ± 3.9 vs. 15.3 ± 5.7) (Figure 3A). Age-stratified seroprevalence analysis demonstrated differential seroconversion patterns: over 67% of younger participants exhibited AAV5/8/9 NAb titers below 40, compared to 56.2% for AAV2 within the 40–160 range. Middle-aged populations showed elevated responses, with over 60% of participants exhibiting AAV5/8/9 NAb titers below 40, compared to AAV2 seroprevalence at 160–640 was 9% higher than in younger participants. Elderly cohorts maintained AAV8/9 titers below 640, while 80% showed AAV5/9 titers below 40, contrasting with 71.4% retaining AAV2 titers at 40–160 (Figure 3C).

Parallel assessments using ARPE-19 cellular models corroborated these age-related trends (Figure 3B). The 40–60 years cohort displayed elevated AAV2 (GMT 83.8 ± 2.6) and AAV9 (20.8 ± 4.1) neutralization compared to younger (GMT AAV2 78.1 ± 3.1, AAV9 12.7 ± 5.4) and older (GMT AAV2 73.9 ± 2.6, AAV9 13.5 ± 4.3) groups. Serological stratification revealed 64% of younger participants exhibited AAV5/8/9 titers < 40 versus 57.1% with AAV2 titers at 40–160. Serological profiling of middle-aged participants revealed that over 58.0% exhibited anti-AAV5/8/9 neutralizing antibody titers below 40, while a comparable proportion (58.0%) demonstrated AAV2-specific titers within the 40–160 range. Elderly populations maintained AAV5/8/9 titers < 640 (100%), while 76.2% retained AAV2 titers within 40–160 (Figure 3D).

### 3.3. Sex-Specific Neutralizing Antibody Prevalence

**Figure 4:**
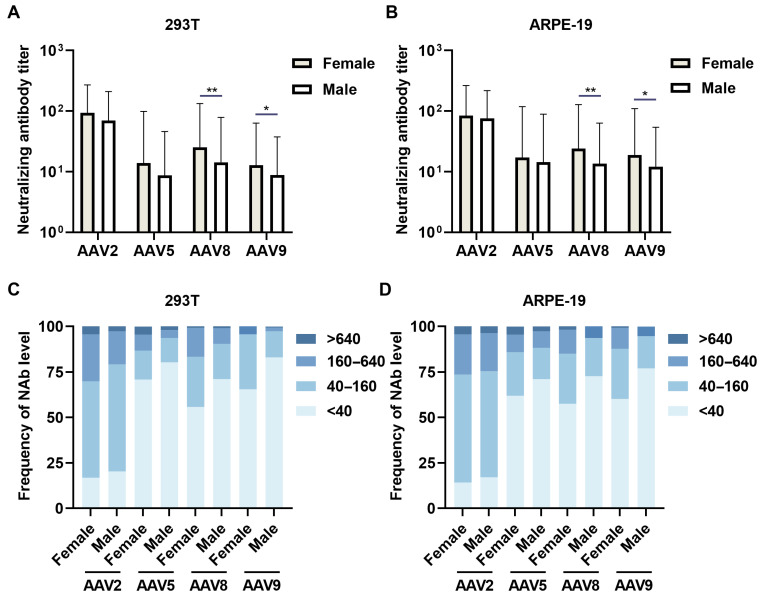
Sex-stratified NAb profiles. Bar graphs illustrate a comparative analysis of sex-specific NAb prevalence between 118 female and 182 male serum donors against wild-type AAV2, AAV5, AAV8, and AAV9. Neutralization activity was quantified in 293T cells (**A**) and ARPE-19 cells (**B**), demonstrating sexually dimorphic antibody responses across serotypes. (**C**,**D**) Histograms present comparative data for AAV2, AAV5, AAV8, and AAV9 corresponding to the experimental results shown in (**A**,**B**), systematically illustrating the sex-stratified distribution patterns of serum NAb against each AAV serotype. *P*-value calculated between female and male groups by unpaired *t*-test; data are presented as GMT with GSD; * *p* < 0.05; ** *p* < 0.01.

A comparative analysis of NAb prevalence across 113 female and 187 male serum specimens revealed sex-dependent immunological profiles. In 239T cellular assays, female-derived sera demonstrated elevated NAb activity against all serotypes (AAV2 GMT 93.4 ± 2.9 vs. male 69.6 ± 3.0, *p* = 0.0832; AAV5 13.9 ± 7.1 vs. 8.6 ± 5.3, *p* = 0.3319; AAV8 25.3 ± 5.3 vs. 14.3 ± 5.5, *p* = 0.1946; AAV9 12.8 ± 5.0 vs. 8.7 ± 4.3, *p* = 0.6295) (Figure 4A). Cross-sex serological profiling demonstrated GMT below 40 against AAV5/8/9 in 55.8–82.9% of individuals, while revealing comparable intermediate-range GMT (40–160) prevalence rates (~50%) for AAV2 in both sexes, indicative of conserved humoral immunity patterns across demographic strata (Figure 4C).

ARPE-19 cellular models revealed statistically significant sex-based differential neutralization for AAV8 (female GMT 23.9 ± 5.3 vs male 13.6 ± 4.7, *p* < 0.01) and AAV9 (18.9 ± 5.8 vs. 12.1 ± 4.5, *p* < 0.05), with female superiority extending to AAV2 (84.3 ± 3.1 vs. 76.1 ± 2.9) and AAV5 (17.1 ± 6.9 vs. 14.3 ± 6.2) (Figure 4B). Platform comparison demonstrated enhanced AAV9 detection in ARPE-19 versus 293T systems (female GMT 18.9 ± 5.8 vs. 12.8 ± 5.0, *p* < 0.05), paralleled by elevated AAV5 titers (17.1 ± 6.9 vs. 13.9 ± 7.1). Seroprevalence stratification maintained consistent patterns: 60.2% of females versus 71.1% of males showed AAV5/8/9 titers below 40, while 59.3% of females and 58.3% of males exhibited AAV2 titers at 40-160 (Figure 4D).

### 3.4. Cell Platform-Dependent AAV Neutralization Serotype Correlations

**Figure 5:**
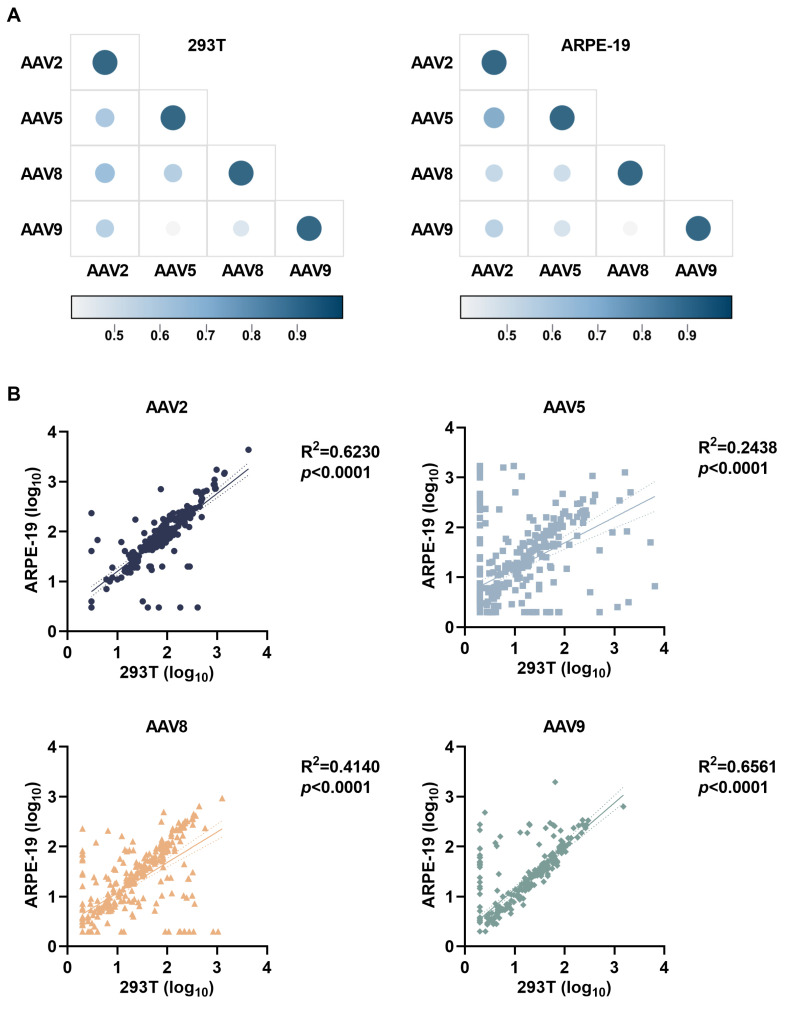
Inter-serotype cross-reactivity profiling of AAV neutralizing antibodies. (**A**) Serotype correlations of neutralizing antibody titers against AAV2, AAV5, AAV8, and AAV9 in 293T or ARPE-19 cells. Correlation coefficient range: R^2^ = 0.4–1.0. (**B**) Cell type-related correlation analysis of neutralizing antibody titers against AAV2, AAV5, AAV8, and AAV9 in 293T and ARPE-19 cellular systems. *p*-value calculated between two cell types by unpaired *t*-test; data are presented as mean with 95% confidence bands.

A comparative analysis of neutralizing antibody profiles across 293T and ARPE-19 cellular systems revealed robust inter-serotype correlations for AAV8/9 (R^2^ = 0.65–0.76) with substantially diminished cross-reactivity in AAV5 (R^2^ = 0.40–0.70), wherein the retinal-derived ARPE-19 platform demonstrated enhanced AAV2-AAV8 cross-neutralization correlations versus 293T counterparts alongside reduced AAV5-AAV9 immune interconnectivity (Figure 5A). Significantly elevated correlation coefficients for AAV2/8/9 neutralization responses relative to AAV5 across both platforms (Figure 5B) underscore the imperative of physiologically relevant retinal models for clinical serotype selection in ocular gene therapy development, particularly for optimizing retinal–tropic vector design through preclinical immunogenicity profiling.

## 4. Discussion

Pre-existing anti-AAV NAbs substantially impair gene transfer efficacy by mediating vector neutralization, with high seroprevalence rates and significant cross-reactivity among serotypes necessitating systematic pre-screening of clinical trial participants. Standardized measurement of neutralization titers against therapeutic serotypes is critical to mitigate NAb-mediated therapeutic attenuation and ensure patient stratification accuracy. This study highlights the importance of choosing the optimal AAV vectors for ocular gene therapy by comparing anti-AAV NAb activity in ARPE-19 retinal cells and 293T kidney cells using human serum. While AAV-mediated retinal gene delivery shows promise for treating inherited retinal diseases, pre-existing NAbs pose a significant challenge. Our findings indicate higher AAV2/5/9 NAb activity in ARPE-19 cells compared to 293T cells, with unique serotype-specific neutralization patterns in retinal models, emphasizing the limitations of traditional neutralization assessments using non-ocular systems.

Standardized NAb detection assays for ocular gene therapy primarily utilize 293T cells. Despite 100-fold higher transgene expression in 293T versus ARPE-19 cells (MOI 10^5^), pre-incubation ensures comparable viral exposure across cell types. Consequently, NAb titers show no significant differences between high-MOI ARPE-19 (10^5^) and low-MOI 293T (10^3^) platforms, supporting methodological consistency for ophthalmic applications (Supplementary Figure S1). A comparative assessment of cellular neutralization hierarchies revealed conserved serotype ranking patterns (AAV2 > AAV8 > AAV5 > AAV9) across ARPE-19 and 293T platforms, while quantifying substantial cellular platform-specific variations in neutralization potency, with ARPE-19 models exhibiting significantly enhanced neutralization against AAV5/9 (42–48% increase) compared to 293T systems. These findings collectively demonstrate the critical cell type-dependent modulation of AAV serotype immune evasion capacity, providing mechanistic insights into cellular platform selection for optimizing the preclinical evaluation of immune escape strategies in gene therapeutic development.

AAV infection in certain cells depends on auxiliary cofactors like laminin receptors and epidermal growth factor receptor, which enhance AAV2 transduction efficiency [20]. Studies show that neutralizing epitopes are often located in coreceptor interaction regions, not just glycan-binding domains [21]. This suggests that varying receptor expression on RPE cells might explain differences in neutralizing antibody levels against AAV vectors. This finding is crucial for improving gene therapy vectors, as preclinical data from non-target models may not accurately predict AAV vectors’ immune evasion in retinal tissues.

This study confirmed the model’s predictive ability in cellular settings and high-lighted the effectiveness of retinal-targeted gene delivery, with age- and sex-related differences noted in the ARPE-19 cell model. No significant age-related prevalence of AAV NAb was found in two cellular platforms. The bell-shaped seroprevalence curve for AAV2 in the middle-aged cohort was corroborated by both platforms. While some studies suggest age-related increases in AAV-specific antibodies [22,23,24], this is consistently observed in AAV8/9. A small study of healthy Chinese participants across different age groups found no significant differences in NAb prevalence against AAV2, AAV3, AAV8, or AAVLK03 [25]. Additionally, female subjects demonstrated higher anti-AAV NAb levels than males in two models, aligning with documented sexual dimorphism in antiviral humoral responses [26,27]. Female-derived specimens exhibited significantly elevated neutralizing antibody titers against AAV8/9 in both 293T and ARPE-19 systems, confirming conserved sex-dimorphic anti-AAV immunity across platforms. Notably, ARPE-19 cellular models demonstrated relative superiority in detecting anti-AAV5/9 neutralizing antibodies within female populations and AAV2/5/9 antibodies in male cohorts compared to 293T systems, revealing cell model-dependent and sex-specific serotype detection sensitivity variations.

The conserved amino acid sequences in AAV capsid proteins drive cross-reactive antibody responses across multiple serotypes due to structural homology [7]. Comparative analysis of neutralizing antibody profiles across 293T and ARPE-19 cellular platforms revealed robust inter-serotype correlations for AAV8/9, indicative of shared conformational epitopes within phylogenetically conserved capsid regions [28], in contrast to the limited AAV5 cross-reactivity linked to its distinct surface glycan architecture and epitope masking mechanisms [9]. Retinal-derived ARPE-19 models demonstrated enhanced AAV2-AAV8/9 cross-neutralization correlations, highlighting tissue-specific microenvironmental regulation of immunogenic epitope exposure. These data advocate for tissue-specific preclinical models like ARPE-19 to enhance clinical outcome predictions and support combinatorial vector strategies leveraging AAV2/8/9’s conserved immunogenic profiles to circumvent pre-existing immunity in ocular gene therapies.

However, as an immortalized cell line, ARPE-19 exhibits structural and functional disparities compared to primary RPE cells [29], potentially overestimating antibody neutralization efficiency. Physiologically, ARPE-19 cells express retinal-specific receptors and exhibit polarized morphology, resembling the retina more closely than standard cell lines [30]; however, their permissiveness to AAV transduction is lower. Conversely, 293T cells, while enabling high-titer viral production and sensitive NAb detection, lack these tissue-specific attributes, risking false-negative/-positive interpretations for ocular applications. Additionally, optimizing the AAV genome—through strategies such as constructing self-complementary vectors to bypass second-strand synthesis for accelerated expression, modifying promoters (substituting CAG for CMV), or inserting tissue-specific enhancers—holds promise for achieving higher transduction efficiency and detection sensitivity in retina-relevant cell lines. Such advancements would significantly enhance clinical translation prospects.

## 5. Conclusions

In summary, this investigation elucidates critical differences in AAV neutralization patterns between retinal and systemic cellular models, establishing that pre-existing NAbs exhibit elevated titers in human retinal epithelium compared to standard 293T assays. The identification of serotype-specific neutralization hierarchies provides a molecular rationale for optimizing vector selection in ocular gene therapy.

## Figures and Tables

**Table 1:** Participant demographics.

Variables	Tested		
Age group (years)	<40	40–60	>60
Overall (N = 300)	210	69	21
Mean	28	51	64
Min, max	18, 40	41, 60	61, 70
Biological sex	Female	Male	
Overall (N = 300)	113	187	

**Table 2:** Neutralizing antibody titers against AAV serotypes: comparative assessment using ARPE-19 and HEK293T cellular models.

AAV Serotypes	AAV2	AAV5	AAV8	AAV9
293T	77.8 ± 3.0	10.3 ± 6.0	17.7 ± 5.5	10.1 ± 4.6
ARPE-19	79.1 ± 3.0	15.3 ± 6.5	16.8 ± 5.0	14.3 ± 5.0
ARPE-19/293T	1.02	1.48	0.95	1.42
*p*	ns	****	*	****

*p*-value calculated between two groups by paired *t*-test; data are presented as GMT with GSD (*n* = 300); ns *p* > 0.05; * *p* < 0.05; **** *p* < 0.0001.

## Data Availability

The dataset generated in this study is available upon reasonable request from the corresponding author.

## Supplementary Materials

The following supporting information can be downloaded at https://www.mdpi.com/article/10.3390/v17070988/s1. Figure S1: AAV2 neutralizing antibody levels are unaffected by MOI variation in 293T Cells.

## References

[1] Wang J.H., Zhan W., Gallagher T.L., Gao G. (2024). Recombinant adeno-associated virus as a delivery platform for ocular gene therapy: A comprehensive review. Mol. Ther..

[2] Le Meur G., Lebranchu P., Billaud F., Adjali O., Schmitt S., Bezieau S., Pereon Y., Valabregue R., Ivan C., Darmon C. (2018). Safety and Long-Term Efficacy of AAV4 Gene Therapy in Patients with RPE65 Leber Congenital Amaurosis. Mol. Ther..

[3] Michaelides M., Besirli C.G., Yang Y., TAC D.E.G., Wong S.C., Huckfeldt R.M., Comander J.I., Sahel J.A., Shah S.M., Tee J.J.L. (2024). Phase 1/2 AAV5-hRKp.RPGR (Botaretigene Sparoparvovec) Gene Therapy: Safety and Efficacy in RPGR-Associated X-Linked Retinitis Pigmentosa. Am. J. Ophthalmol..

[4] Li R., Jing Q., She K., Wang Q., Jin X., Zhao Q., Su J., Song L., Fu J., Wu X. (2023). Split AAV8 Mediated ABCA4 Expression for Gene Therapy of Mouse Stargardt Disease (STGD1). Hum. Gene Ther..

[5] Murray S.J., Russell K.N., Melzer T.R., Gray S.J., Heap S.J., Palmer D.N., Mitchell N.L. (2021). Intravitreal gene therapy protects against retinal dysfunction and degeneration in sheep with CLN5 Batten disease. Exp. Eye Res..

[6] Kruzik A., Fetahagic D., Hartlieb B., Dorn S., Koppensteiner H., Horling F.M., Scheiflinger F., Reipert B.M., de la Rosa M. (2019). Prevalence of Anti-Adeno-Associated Virus Immune Responses in International Cohorts of Healthy Donors. Mol. Ther. Methods Clin. Dev..

[7] Boutin S., Monteilhet V., Veron P., Leborgne C., Benveniste O., Montus M.F., Masurier C. (2010). Prevalence of serum IgG and neutralizing factors against adeno-associated virus (AAV) types 1, 2, 5, 6, 8, and 9 in the healthy population: Impli-cations for gene therapy using AAV vectors. Hum. Gene Ther..

[8] Mingozzi F., High K.A. (2011). Therapeutic in vivo gene transfer for genetic disease using AAV: Progress and challenges. Nat. Rev. Genet..

[9] Louis Jeune V., Joergensen J.A., Hajjar R.J., Weber T. (2013). Pre-existing anti-adeno-associated virus antibodies as a chal-lenge in AAV gene therapy. Hum. Gene Ther. Methods.

[10] Schulz M., Levy D.I., Petropoulos C.J., Bashirians G., Winburn I., Mahn M., Somanathan S., Cheng S.H., Byrne B.J. (2023). Binding and neutralizing anti-AAV antibodies: Detection and implications for rAAV-mediated gene therapy. Mol. Ther..

[11] D’Alessio A.M., Boffa I., De Stefano L., Soria L.R., Brunetti-Pierri N. (2024). Liver gene transfer for metabolite detoxification in inherited metabolic diseases. FEBS Lett..

[12] Reichel F.F., Peters T., Wilhelm B., Biel M., Ueffing M., Wissinger B., Bartz-Schmidt K.U., Klein R., Michalakis S., Fischer M.D. (2018). Humoral Immune Response After Intravitreal But Not After Subretinal AAV8 in Primates and Patients. Investig. Ophthalmol. Vis. Sci..

[13] Bennett J., Wellman J., Marshall K.A., McCague S., Ashtari M., DiStefano-Pappas J., Elci O.U., Chung D.C., Sun J., Wright J.F. (2016). Safety and durability of effect of contralateral-eye administration of AAV2 gene therapy in patients with childhood-onset blindness caused by RPE65 mutations: A fol-low-on phase 1 trial. Lancet.

[14] Desrosiers M., Dalkara D. (2018). Neutralizing Antibodies Against Adeno-Associated Virus (AAV): Measurement and Influence on Retinal Gene Delivery. Methods Mol. Biol..

[15] Meyer N.L., Chapman M.S. (2022). Adeno-associated virus (AAV) cell entry: Structural insights. Trends Microbiol..

[16] Kaludov N., Brown K.E., Walters R.W., Zabner J., Chiorini J.A. (2001). Adeno-associated virus serotype 4 (AAV4) and AAV5 both require sialic acid binding for hemagglutination and efficient transduction but differ in sialic acid linkage specificity. J. Virol..

[17] Shen S., Bryant K.D., Brown S.M., Randell S.H., Asokan A. (2011). Terminal N-linked galactose is the primary receptor for adeno-associated virus 9. J. Biol. Chem..

[18] Summerford C., Samulski R.J. (1998). Membrane-associated heparan sulfate proteoglycan is a receptor for adeno-associated virus type 2 virions. J. Virol..

[19] Cehajic-Kapetanovic J., Le Goff M.M., Allen A., Lucas R.J., Bishop P.N. (2011). Glycosidic enzymes enhance retinal transduction following intravitreal delivery of AAV2. Mol. Vis..

[20] Nakamura H., Tanaka T., Zheng C., Afione S.A., Warner B.M., Noguchi M., Atsumi T., Chiorini J.A. (2022). Correction of LAMP3-associated salivary gland hypofunction by aquaporin gene therapy. Sci. Rep..

[21] Large E.E., Chapman M.S. (2023). Adeno-associated virus receptor complexes and implications for adeno-associated virus immune neutralization. Front. Microbiol..

[22] Klamroth R., Hayes G., Andreeva T., Gregg K., Suzuki T., Mitha I.H., Hardesty B., Shima M., Pollock T., Slev P. (2022). Global Seroprevalence of Pre-existing Immunity Against AAV5 and Other AAV Serotypes in People with Hemophilia A. Hum. Gene Ther..

[23] Calcedo R., Morizono H., Wang L., McCarter R., He J., Jones D., Batshaw M.L., Wilson J.M. (2011). Adeno-associated virus antibody profiles in newborns, children, and adolescents. Clin. Vaccine Immunol..

[24] Ertl H.C.J. (2021). T Cell-Mediated Immune Responses to AAV and AAV Vectors. Front. Immunol..

[25] Ling C., Wang Y., Feng Y.L., Zhang Y.N., Li J., Hu X.R., Wang L.N., Zhong M.F., Zhai X.F., Zolotukhin I. (2015). Prevalence of neutralizing antibodies against liver-tropic adeno-associated virus serotype vectors in 100 healthy Chinese and its potential relation to body constitutions. J. Integr. Med..

[26] Liu Q., Huang W., Zhao C., Zhang L., Meng S., Gao D., Wang Y. (2013). The prevalence of neutralizing antibodies against AAV serotype 1 in healthy subjects in China: Implications for gene therapy and vaccines using AAV1 vector. J. Med. Virol..

[27] Dhungel B.P., Winburn I., Pereira C.D.F., Huang K., Chhabra A., Rasko J.E.J. (2024). Understanding AAV vector im-munogenicity: From particle to patient. Theranostics.

[28] Gardner M.R., Mendes D.E., Muniz C.P., Martinez-Navio J.M., Fuchs S.P., Gao G., Desrosiers R.C. (2022). High con-cordance of ELISA and neutralization assays allows for the detection of antibodies to individual AAV serotypes. Mol. Ther. Methods Clin. Dev..

[29] O’Leary F., Campbell M. (2023). The blood-retina barrier in health and disease. FEBS J..

[30] Finnemann S.C., Bonilha V.L., Marmorstein A.D., Rodriguez-Boulan E. (1997). Phagocytosis of rod outer segments by retinal pigment epithelial cells requires alpha(v)beta5 integrin for binding but not for internalization. Proc. Natl. Acad. Sci. USA.

