# Supplementary material for: Retinal Epithelial Neutralization Assay Optimizes AAV Serotype Selection for Ocular Gene Therapy
Source: Viruses. 2025 Jul 15;17(7):988. doi: 10.3390/v17070988 (PMC12300778; doi:10.3390/v17070988)
Supplement: Supplementary file 1 [file viruses-17-00988-s001.zip › viruses-3700122-supplementary.pdf]

Article

Retinal Epithelial Neutralization Assay Optimizes AAV Serotype Selection for Ocular Gene Therapy

Yao Li, Yujia Chen, Nan Huo, Zuyuan Jia, He Huang, Zhenghao Zhao *, Shipo Wu * and Lihua Hou *

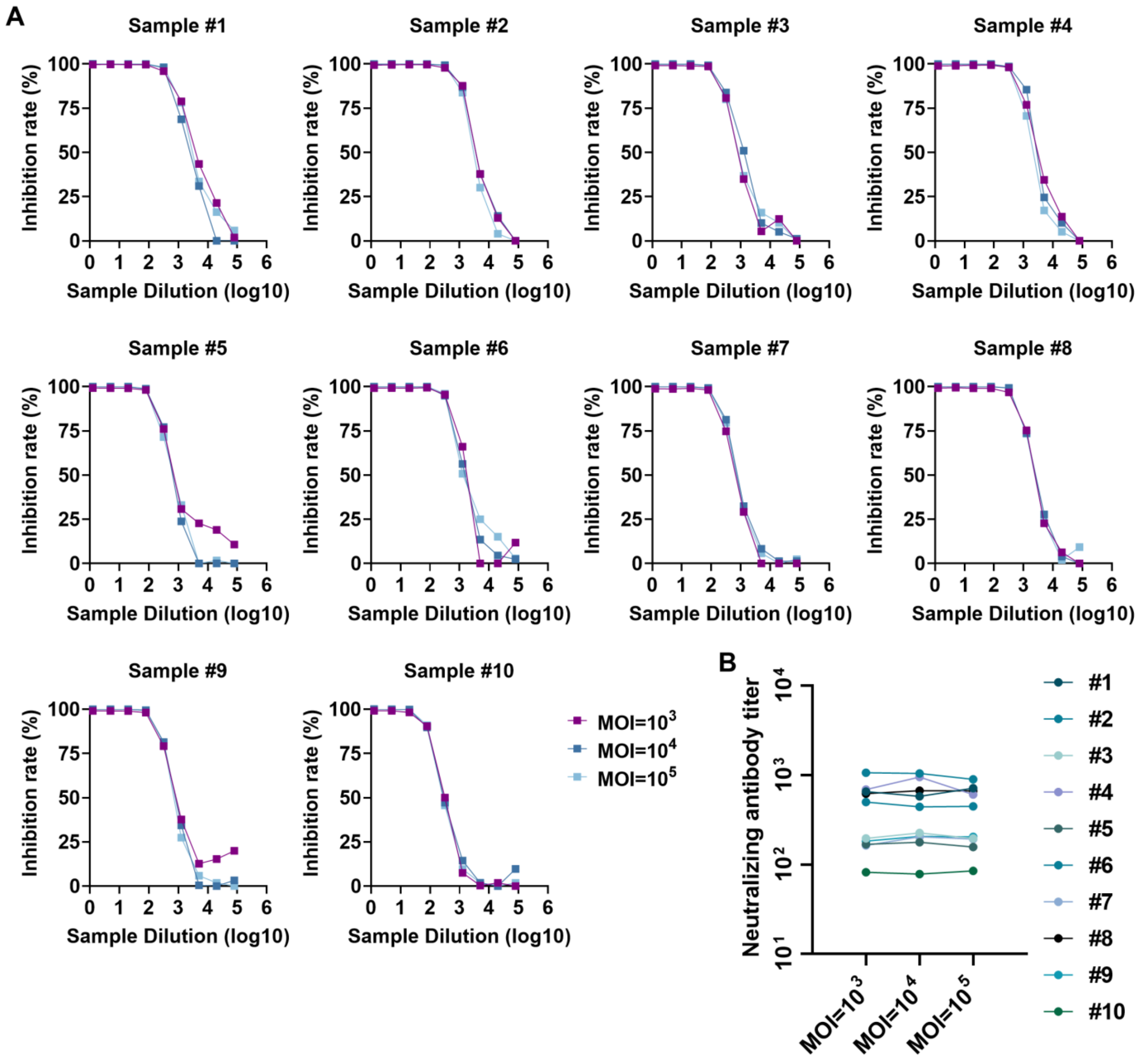

Supplementary Figure 1. AAV2 neutralizing antibody levels are unaffected by MOI variation in 293T Cells. (A) Inhibition rates against AAV2 across MOIs 10^3–10^5 in 10 serum samples. (B) Consistent NAb titers under tested multiplicities of infection. Results indicate MOI-independent NAb stability within this range (n=10).
